# Benefits of Aerobic Exercise for Breast Cancer Survivors: A Systematic Review of Randomized Controlled Trials 

**DOI:** 10.31557/APJCP.2019.20.11.3197

**Published:** 2019

**Authors:** Amira Hassan Bekhet, Ahmed Ramadan Abdalla, Horeya M Ismail, Doaa M Genena, Nermin A Osman, Ayman El Khatib, Rami Labib Abbas

**Affiliations:** 1 *Faculty of Physical Therapy, Cairo University, *; 2 *Medical Research Group of Egypt Cairo, *; 3 *Faculty of Medicine, Mansoura University, Mansoura, *; 4 *Cancer Research Cluster, *; 5 *Medical Research Institute, Alexandria, University, Alexandria, Egypt, *; 6 *Department of Physical Therapy, Faculty of Health Sciences, Beirut Arab University, Beirut, Lebanon. *

**Keywords:** Aerobic exercise, breast cancer, physical activity, quality of life

## Abstract

**Background:**

Physical exercise may be beneficial to breast cancer (BC) survivors. Here, we systematically summarized the effects of aerobic exercise in BC survivors. We conducted a systematic review of randomized controlled trials (RCTs).

**Methods:**

We searched PubMed, Web of knowledge, Scopus, Cochrane Central, Virtual Health Library and PEDRO databases for relevant RCTs, comparing aerobic exercise with usual care among BC survivors. Data were extracted and evidence was synthesized narratively.

**Results:**

Twelve studies were included in this systematic review. Studies reported that aerobic exercise can significantly improve the quality of life in BC survivors. Moreover, aerobic exercise alleviated the symptoms of depression and anxiety. However, current evidence from the included studies showed that there was no significant benefit for aerobic exercise in terms of weight loss. **Conclusion: **Our study suggests that aerobic exercise is beneficial to BC survivors.

**Clinical Relevance:**

Aerobic exercise should be recommended in the therapeutic and rehabilitative regimens of BC survivors.

## Introduction

Breast cancer (BC) is the second most common cancer worldwide and the most frequent cancer among women (25% of the overall cancer among women) with an increasing incidence in the last decade. The World Health Organization (WHO) ranked BC as the fifth most common cause of cancer mortality worldwide (522,000 deaths in 2012) (Torre et al., 2015). The significant improvements in BC screening, diagnosis, and treatment over the past few decades led to a decline in BC-related death rate, which in turn caused a substantial increase in the number of BC survivors (Siegel et al., 2017). 

Physical activity plays a significant role in improving quality of life (QoL), physical, psychological functions, and other health indicators in cancer patients (Fong et al., 2012). The literature showed several benefits for exercise therapy as a supportive care for BC patients and survivors (Brockow et al., 2016; Kim et al., 2009; McNeely et al., 2006a) These beneficial effects vary according to disease stage, type of primary treatment and the lifestyle of patients (Knols et al., 2005) 

The survival outcome in BC survivors is likely to have some correlation with exercise; long-term physical activity levels are important for BC prognosis and are associated with improved survival (Bertram et al., 2011). Moreover, previous research showed that exercise improves the QoL and lessens the symptoms of depression and anxiety in BC survivors (Segar et al., 1998). Additionally, it was associated with benefits on muscle strength and body composition (Zhu et al., 2016). Therefore, the American Cancer Society guidelines recommend at least 150 minutes on a weekly basis for exercise practicing. These guidelines also encouraged long-term adherence to daily exercise beyond the initial post-surgical rehabilitation (Rock et al., 2012). 

Aerobic exercise is a form of physical activity that depends primarily on the aerobic energy-generating process i.e. it requires free oxygen to meet the demands of aerobic metabolism. It involves running/jogging, swimming, cycling, and walking. Several randomized controlled trials (RCTs) suggested the efficacy of aerobic exercise in reducing cancer-related fatigue among BC survivors (Cantarero-Villanueva et al., 2013; Carter et al., 2016; Vardar Yağlı et al., 2015). Moreover, aerobic physical activity was found effective in improving anthropometric measures as body weight, body composition, and VO2 peak, as well as reducing inflammatory markers (Jones et al., 2013; Matthews et al., 2007; Tizdast et al.,2016)..

The benefits of exercise in general in BC survivors have been assessed in several systematic reviews and meta-analyses (Floyd and Moyer, 2010; Meneses-Echávez et al., 2015; Zhu et al., 2016). However, there is a gap in addressing the effects of specific exercise types in BC rehabilitation program as published trials showed conflicting results, especially in terms of physical activity and QoL outcomes. According to our knowledge, no former systematic review or meta-analysis has assessed the benefits of aerobic exercise specifically in BC survivors. The National Comprehensive Cancer Network (NCCN) reported the need for future research work to identify the effects of specific modes of exercise on specific outcomes as fatigue and QoL in cancer survivors (Schmitz et al., 2010).

We performed this systematic review of the literature to investigate the efficacy of aerobic exercise intervention on physical activity, QoL (primary outcomes), weight, inflammatory markers and sleep among BC survivors by scrutinizing the published trials.

## Materials and Methods

We followed the PRISMA statement guidelines (Table S1 of Supplementary material) during the preparation of this review (Moher et al., 2009). This systematic review is registered on the PROSPERO international prospective register of systematic reviews (CRD42017060106).

*Literature search strategy*

We searched PubMed https://www.ncbi.nlm.nih.gov/pubmed/, Web of knowledge http.apps.webofknowledge.com, Scopus https-www-scopus.com, Cochrane Central https://www.cochranelibrary.com/central, Virtual Health Library (VHL) https://bvsalud.org/en/ and PEDRO https://www.pedro.org.au/ for RCTs that compared Aerobic Exercise with usual care in BC survivors. We used the following search strategy “(breast cancer survivors* OR breast tumor survivors*) AND (Aerobic exercise OR physical fitness OR physical therapy OR rehabilitation)”. No restrictions by publication period were employed.. In addition, we manually scanned the references list of selected articles for relevant studies.

*Eligibility criteria and study selection*

We included RCTs that met our inclusion criteria, a) Population: BC survivor women, treated with chemotherapy or radiotherapy (stopped at least six months earlier) or patients who have started adjuvant endocrine therapy (antiestrogens, aromatase inhibitors, LHRH agonists, or combinations) not less than 4 months earlier and not scheduled for and not currently undergoing chemo-/radiation therapy, b) Intervention: Studies in which the intervention was aerobic exercise (walking, cycle ergometers, swimming, and stair climbing), not mixed with any other type of exercise, c) Comparator: Studies where the control group received usual care, d) Outcomes: Physical Activity level, QoL, Sleep parameters, weight, as well as inflammatory markers.

We excluded studies with the following criteria: a) patients on active therapy, b) patients with uncontrolled cardiac or vascular disease, c) patients exercising at a regular basis at baseline, and d) non-English articles. The retrieved citations were added to a Microsoft Excel sheet that was distributed to reviewers. Eligibility screening was done through two separate steps: a) titles and abstracts screening, and b) full texts screening. Each study title and/or full-text was screened by two independent reviewers (therefore, each reviewer screened half the retrieved citations) and disagreements were resolved by discussion between all reviewers.

*Data Extraction*

Three independent reviewers extracted the relevant data from the included studies, using a preformatted data extraction sheet. Disagreements were resolved by the opinion of a fourth reviewer. The extracted data included: a) baseline characteristics of participants as age and cancer stage, b) general characteristics of included studies as design, sample size, and assessed outcomes, c) characteristics of the intervention as type, intensity, and duration and d) the results of each included study. The summary effect size was difficult to calculate due to the lack of sufficient data for subsequent pooling, as well as lack of the homogeneity needed to make a calculation.

*Risk of bias assessment*

 The risk of bias in included RCTs was assessed according to the Cochrane Handbook for Systematic Reviews of Interventions 5.1.0 (updated March 2011) using the risk of bias assessment table, provided in the same book (part 2, Chapter 8.5). The Cochrane risk of bias assessment tool includes the following domains: Random sequence generation (selection bias), allocation sequence concealment (selection bias), blinding of participants and personnel (performance bias), blinding of outcome assessment (detection bias), incomplete outcome data (attrition bias), selective outcome reporting (reporting bias), and other potential sources of bias. The reviewer judgment is categorized as low risk, high risk, or unclear risk of bias. For risk of bias exclusion across the included studies, we compared the reported outcomes between the studies to exclude reporting of selective outcome.

## Results

*Literature search results*

**Figure 1:**
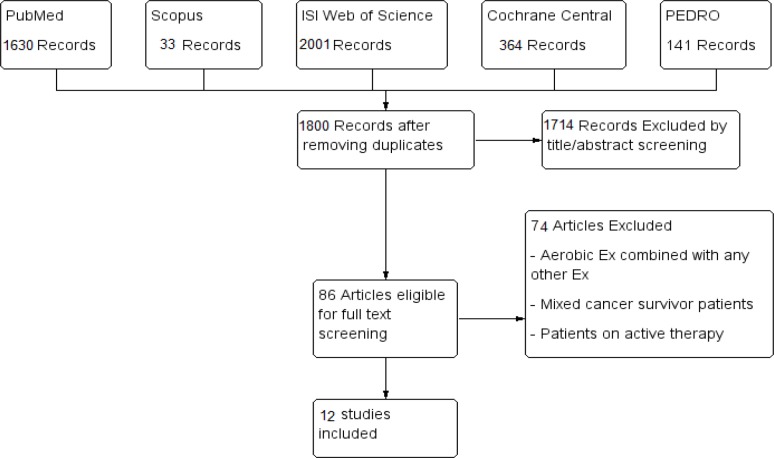
PRISMA Flow Diagram of Screening and Study Selection Process

We retrieved 1,800 unique citations. After the initial title and abstract screening, 86 full-text articles were retrieved and screened for eligibility. After full text screening, 12 RCTs (n= 1,120 patients) were included in our systematic review (see PRISMA flow diagram; Figure 1).

*Characteristic of included studies*

The sample size of included studied ranged from 19 to 357 patients, with an average age of 44 to 61 years. Aerobic exercise was the only intervention of interest in this meta-analysis either supervised or home-based training programs. Most of the included studies reported walking as the preferred type of activity among BC survivors. Exercise sessions were of moderate intensity. Irwin et al., (2008), Matthews et al., (2007) and Saarto et al., (2012) assessed physical activity outcome, while Campbell et al., (2017), Murtezani et al., (2014) and Saarto et al., (2012) examined the effect of aerobic exercise on the QoL. Murtezani et al., (2014), Roveda et al., (2016) and Tizdast et al., (2016) investigated the role of aerobic exercise on weight reduction among BC survivors. Two studies assessed the effect of aerobic exercise on inflammatory markers (Fairey et al., 2005; Jones et al., 2013). Only one study reported the effect of aerobic exercise on sleep parameters (Roveda et al., 2016). A summary of the design and main findings of included studies is shown in Table 1, and the baseline characteristics of their participants are shown in Table 2.

*Risk of Bias assessment results*

**Figure 2:**
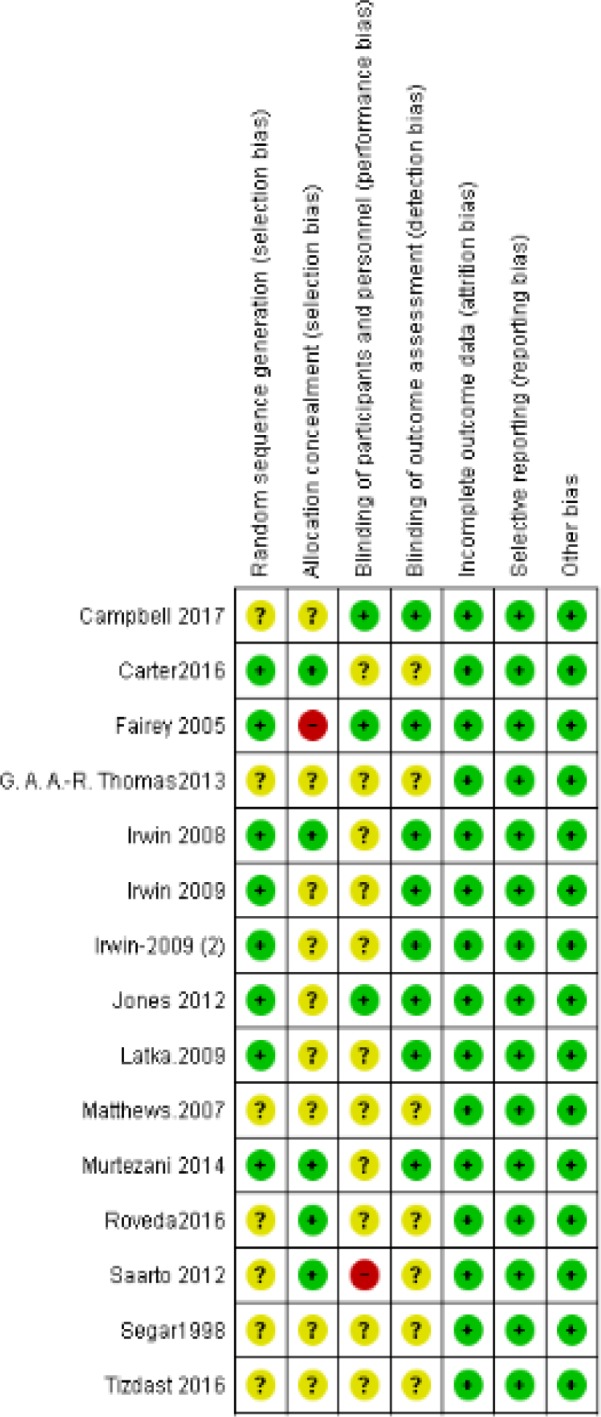
Risk of Bias Summary for Included Studies

We used the Cochrane risk of bias assessment tool to assess the bias of included studies. All included studies were of low risk in terms of selective reporting and attrition bias. Only five studies were of low risk of bias in terms of allocation concealment (Carter et al., 2016; Irwin et al., 2008; Murtezani et al., 2014; Roveda et al., 2016; Saarto et al., 2012), while three studies were of low risk of bias in terms of blinding the participants/personnel (Campbell et al., 2017; Fairey et al., 2005; Jones et al., 2013). The summary of risk of bias assessment is shown in Figure 2, and the reasons for authors’ judgments are shown in Table S2 of Supplementary material.

*Physical Activity*

Mathews et al., (2007) reported higher activity levels in the intervention group compared to usual care group (p ≤0.04). Moreover, Irwin et al., (2008) reported that in the 7-day physical activity log, the exercise group increased from moderate to vigorous intensity reactional activity (129 min per week, p <0.001), compared to a smaller increase in the usual care group (45 min per week). However, Saarto et al., (2012) reported an insignificant difference between the exercise and control groups (P=0.97). They detected a significant linear trend between higher physical activity, improved QoL, and recovery from fatigue.

*Quality of life*

Saarto et al., (2012) measured the QoL using the EORTC-QIQ-C30 or BR-23 forms. They reported no significant difference after 12 months in QoL between exercise and control groups. Campbell et al., (2017) used the FACT-COG tool to measure the cognitive symptoms impacting the QoL, but they found no statistically significant improvement. Murtezani et al., (2014) used the FACT B score to measure the overall QoL after 10 weeks of intervention. They reported that the FACT B score was increased in the intervention group and was decreased in controls (P = 0.003).

*Weight*

Tizdast et al., (2016) reported that higher weight reduction was achieved in the interval exercise group (P=0.012). Murtezani et al., (2014) and Fairy et al., (2005) did not observe any significant difference between the exercise and control groups in terms of weight loss (95% CI,-4.8 to 2.25 Kg; P=0.47 and 95% CI,-1.6 to 0.6 Kg; P=0.39, respectively). On the contrary, Roveda et al., (2016) found that the control group had a significant weight reduction after 3 months of physical activity (P= 0.01).

*Inflammatory markers*

Two studies examined the effects of aerobic exercise on inflammatory makers in BC survivors. Fairy et al., (2005) reported that C-reactive protein (CRP) decreased by 139 mg/l in the exercise group, while it increased by 0.1 mg/l in the control group. However, this change was not significant (95% CI,-3.09 to 0.1 mg/l; P=0.66) Moreover, Jones et al. (2013) reported an insignificant difference between randomization groups regarding serum concentrations of interleukin (IL)-6, CRP, and tumor necrosis factor (TNF-α).

*Sleep parameters*

Only one study reported the effects of exercise on sleep parameters for BC survivors. Roveda et al., (2016) found sleep efficiency (SE) to be significantly decreased in the control group, it but remained unchanged in the exercise group (P=0.001). The mean activity score (MAS) was significantly increased in the control group, but it remained unchanged in the exercise group (p <0.001). The authors reported a significant decrease (P=0.03) in the actual sleep time (AST) and a significant increase (P=0.02) in the movement fragmentation index (MFI) after aerobic exercise intervention, compared to baseline scores. 

**Table 1:** Shows a Summary of the Design and Main Findings of Included Studies

study ID	Population (cancer stage)	Mean age (SD) Intervention/control	sample size intervention/control groups	duration of treatment	Type of Exercise Intervention/control	intensity	frequency	Outcomes measured	Summary of results
Matthews 2007	Stage I–III breast cancer	51.3 (9.0) / 56.9 (12.3)	22 / 14	12 weeks	Walking / Usual care	Moderate	*In the first 4 weeks, three times/week. *5–7 weeks, four times/week *The final 5 weeks, five times/week	* Overall physical activity level measured by CHAMPS questionnaire (MET-h/week) and the actigraph. *Effect of intervention on physical activity behaviors and body composition measured by (body mass, Fat-free mass , fat mass, body fat).	* Intervention proved effective in increasing physical activity levels among breast cancer survivors. *No significant changes in body fat and composition.
Campbell 2017	stage I-IIIA breast cancer	53.2 (7.0) / 51.4 (5.1)	10/9	24 weeks	Walking / usual lifestyle	Moderate-to-vigorous	150 min/week of aerobic exercise + two 45-minute supervised sessions per week in a research gym + two additional 30 minute unsupervised home sessions	*Self-reported cognitive dysfunction and associated impact on quality of life measured by (FACT-Cog). *Objective neuropsychological tests in cancer survivors based on TMT-A and TMT-B, Stroop test, fMRI.	No statistically significant improvement in self-reported cognitive function and neuropsychological tests.
Roveda 2016	NR	55.2 (6.8) / 58.2 (6.4)	19 / 21	3 months	Brisk walking .	Moderate	2 sessions of 1-hour brisk walking per week	*Anthropometric and Body Composition Evaluation *Actigraphic Monitoring of Sleep Patterns *Actigraphic Monitoring of Activity Level and Circadian Rhythm *Armband-Based Monitoring of Energy Expenditure	aerobic PA has a protective effect against factors promoting sleep disruption. PA improved sleep behavior and reduced sleep worsening of BCS.
Fairey 2005	Stage I-IIIA breast cancer	59 (5) / 58 (6)	24 / 28	15 weeks	cycle ergometers / no activity	Moderate	3 times per week	*Effect of PA on CRP * Effect of PA on cardiovascular risk factors (RHR, HRR, SBP, DBP, TC, LDL-C, HDL-C, TG, and TC:HDL-C ratio).	*PA had borderline significant effect on CRP *PA also had a clinically and statistically significant effect on HRR and clinically but not statistically significant effects on RHR, SBP, DBP, HDL-C, and TG
Thomas 2013	Stage 0–111A breast cancer	56.5 (9.8) / 55.1 (7.6)	35 / 30	6 months	Walking / usual care	Moderate intensity	*Three 15-minute sessions (week1) * gradually built up to five 30-minute (Week 5)	Effect of aerobic exercise on metabolic syndrome prevalence - Waist circumference (cm) - HDL-C (mg/dL) -Triglycerides (mg/dL) - Glucose (mg/dL) - Metabolic syndrome z-score	*In overweight or obese, physically inactive BCS the adherence to a moderate intensity aerobic exercise was associated with improvements in metabolic syndrome criteria.
Murtezani 2014		53 ( 11) / 51( 11)	30/32	10 week	Aerobic exercise(treadmills, stationary bicycles, and stairclimbing Machines) / usual care	Moderate intensity	three times per week for 10 weeks	*-Anthropometric Changes a) BMI b) % Total body fat *-Quality of life assessment by (FACTB) *12 min walk test.	Results revealed that 10 week of moderateintensity aerobic exercise program significantly improves QOL and physical functioning in breast cancer survivors
study ID	Population (cancer stage)	Mean age (SD) Intervention/control	sample size intervention/control groups	duration of treatment	Type of Exercise Intervention/control	intensity	frequency	Outcomes measured	Summary of results
Carter 2016	DCIS or stage I-IIIA breast cancer	55(8) for all participants	76/76	3 months	treadmill walking/usual care	Vigrous-Moderate intensity	-goal of meeting ≥150 min of moderate-intensityexercise -less than 30 min of vigorous or 60 min of self-reported moderate- intensity physical activity per week.	-baseline and immediately postintervention (M3) on measures of physical activity (accelerometry), graded walk test, and average fatigue over the previous 7 days. -calculating Pressure product rate (PPR)	Lower rate-pressure product during submaximal walking was significantly associated with reduced fatigue in BCS. for Cancer Survivors Exercise/physical activity training programs that lower the physiological strain during submaximal walking may produce the largest improvements in reported fatigue.
Saarto 2012	Histologically proven invasive breast cancer T1-4,N0-3,M0	Mean age (range) 52.3(36-68)/52.4(35-68)	263/237	12 months	supervised and home training -supervised training: endurance training such as walking, Nordic walking or aerobic training, jumps and leaps. In addition, brisk endurance training (walking, Cycling, swimming etc.)	progressive vigorous exercise training	-supervised training: once a week 60-minute aerobics and circuit training	Changes in quality of life (QoL) during the intervention measured by the * EORTC QLQ-C30 and *BR-23 *FACIT-F BDI questionnaires	*The amount of physical activity increased from baseline to 12 months *Neuromuscular performance improved significantly in the training group *No significant effect of the intervention was observed on cardiorespiratory fitness. *No significant difference was found between the exercise and control groups in changes of QoL during the intervention
Segar 1998	Any type of breast cancer surgery	47.5(7.1)/61.8(8.1)	16/8	10 weeks	Exercise group / Exercise plus Behavior / control group ((study participant choose her favorite and most conveient type of aerobic exercise))	=< 60 of age predicted maximum heart rate	a minimum 30 minutes/seesion, 4 days/week	*Exercise adherence *Depression (Beck depresion Inventory) *Anxiety(spellberger stat trait Anxiety inventory) *Rosenberg selfesteem inventory	*women who exercised had signficantly less depression and state and anxiety over time compared to control *after crossover,the control group demonstrated comperable improvement in both depressive and state anxiety scores, self esteem didn`t changed significantly *subjects wh recieved exercise recommendation from their physcian exercised significantly more than subjects who recieved no recommendation
Jones 2012	stage0 to IIIA	56.4 (9.6)/ 55.4(7.6)	36/32	6 months	Aerobic exercise/ usual care	moderate- intensity aerobic exercise	150 minutes over 3 weeks	1-Arthopometric Changes c) BMI d) % Total body fat 2- Changes in inflammatory markers Levels.	Results revealed that the moderate-intensity aerobic exercise intervention did not signiﬁcantly alter concentrations of IL-6, CRP, or TNF-a.
study ID	Population (cancer stage)	Mean age (SD) Intervention/control	sample size intervention/control groups	duration of treatment	Type of Exercise Intervention/control	intensity	frequency	Outcomes measured	Summary of results
Tizdast 2016	stage I-III breast cancer The Yale Exercise and Survivorship Study.	44.1(4.6) for all participants	continous exercise grpup(n=9), interval exercise group (n=9), control group(n=9)	8 weeks	Continuous exercise group/ Interval exercise group/ control group (planned treadmill exercise))	varied according to trainng schedule	three times per week	*Body weight, *body mass index, *adipose tissue, body fat percentage *Waist to Hip Ratio(WHR)	*The highest reduction in weight, body fat percentage and adipose tissue and the highest increase in VO2 peak occurred in the interval exercise group. *The waist-hip ratio reduced equally in both exercise groups *The anthropometric variables, body fat percentage and adipose tissue decreased significantly in both exercise groups but the differencebetween the two exercise groups was not significant
Irwin 2008	stage 0 (in situ) to stage IIIA breast cancer	56.5 ( 9.5) /55.1 ( 7.7 )	37/38	6 months		Moderate To vigorous intensity	150 min/wk. of supervised gym-based and home-based aerobic exercise. 44 min/wk. among usual-care to maintain current physical activity level.	1-Arthopometric Changes a) BMI b) % Total body fat 2- Satisfaction and competence questionnaire.	Women in the exercise-intervention group increased their average pedometer steps by 1621 steps per day compared with a decrease of 60 steps per day among women in the usual-care group (P <.01).
Irwin 2009 (1)	stage 0 to IIIA breast cancer	56.4 ( 9.5) /55.6 (7.7)	37/38	6 months		moderate-intensity aerobic exercise	150 minutes per week 30 minutes on 5 days	1-Arthopometric Changes c) BMI d) % Total body fat 2- Changes in insulin, IGF-1, IGFBP3 Levels.	Moderate-intensity aerobic exercise, per week, used in the study is proved to be tolerated in breast cancer survivors and efficacious in decreasing levels of IGF-I and IGFBP-3.
Irwin 2009 (2)	Stage 0–IIIA	56.5 (9.5)/ 55.1( 7.7)	37/38	6 months		Moderate intensity aerobic exercise	150 min/week .	1-Arthopometric Changes g) BMI h) % Total body fat 2-Changes in inflammatory markers Levels. (BMD)	Aerobic exercise, such as brisk walking, was associated with favorable changes in body fat and LBM, and maintenance of BMD in postmenopausal breast cancer survivors
Latka 2009	stage 0 to stage IIIA breast cancer	56.5(9.5)/NR	37/38	6 months		Moderate	exercise goal was 30 min of exercise 5 days/week for 6 months. *(Participants wereasked to perform three 15-minsessions during Week1, building to five 30min-sessions by Week5)	*BMI *waist circumference *FACT-B Breast Cancer subscale *Transtheoretical Model Stage	*Women with higherBMI, larger waist circumference, fewer minutes of physical activity at baseline associated with breast cancer as measured by the FACT-B Breast Cancer subscale score had a lower adherence to the exercise prescription. *Specifically, a lower BMI and a higher degree of readiness to change physical activity behavior were associated with better adherence.

**Table 2:** Shows Baseline-Characteristics of Included Studies Participants

study	Study arm	Sample Size Experimental/ control	Age mean (SD)	Disease stage (%) In situ Stage I Stage II Stage IIIA	BMI mean (SD)	Weight (kg) mean (SD)	Menopausal status *Pre-menopausal *Post-menopausal	Time since diagnosis	Ethnicity (%)	Pedometer average, steps/d	Physical activity
Matthews 2007	Experimental	22	51.3 (9)	Stage I (59%) Stage II /III (18%) Not available (23%)	28.3 (4.9)	74.9(14.5)	Post-menopausal	0.9 (0.7 – 1)	White 82% African American/other 18%	7,409.4 (2,791.1)	*By CHAMPS unit of MET-h/week: Total activity 32.7 (24.0)
	control	14	56.9 (12.3)	Stage I (64%) Stage II/III (21%) Not available (14%)	29.9 (7.1)	78.9(20.3)	Post-menopausal	0.7 (0.6 – 1)	White86% African American/other 14%	5,939.00 -2,203.50	*By CHAMPS unit of MET-h/week: Total activity 22.2 (20.7)
Campbell 2017	Experimental	10	53.2 (7)	Stage I: 0 Stage II: 10(100) Stage III:0	26.3 (5.7)	71.1 (15.9)	Post-menopausal	NR	NR	NR	(MET-hr./wk.) 14.1 (12.0)
	control	9	51.4 (5.1)	Stage I: 0 Stage II: 7(78) Stage III: 2(22)	26.1 (5.5)	66.6 (16.7)	Post-menopausal	NR	NR	NR	(MET-hr./wk.) 11.2 (7.0)
Roveda 2016	Experimental	19	55.2 (6.8)	NR	25.4 (3.8)	67.1 (11.9)	Pre & post-menopausal	NR	NR	9790.9 ± 2817.2	physical activity level 1.49 ( 0.10)
	control	21	58.2 (6.4)	NR	24.6 (3.4)	64.5 (9.6)	Pre & post-menopausal	NR	NR	11001.6 ± 3702.2	physical activity level 1.56 ( 0.14)
Fairey 2005	Experimental	24	59 (5)	Stage I (42%) Stage IIa (25%) Stage IIb (25%) Stage IIIa (8%)	29.4 (7.4)	78.1 (20.4)	Post-menopausal	NR	NR	NR	fitness described in baseline values for peak oxygen consumption (ml/kg/min 18.6 (3.9)
	control	28	58 (6)	Stage I (39%) Stage IIa (39%) Stage IIb (18%) Stage IIIa -4%	29.1 (6.1)	79.4 (16.4)	Post-menopausal	NR	NR	NR	cardiopulmonary fitness described in baseline values for peak oxygen consumption (ml/kg/min) 18.8 (3.8)
Thomas 2013	Experimental	35	56.5 (9.8)	In situ (11%) Stage I (54%) Stage II (26%) Stage IIIA -9%	30.8 (5.9)	82.1 (16.5)	Post-menopausal	3.6 (2.2)	White 83% African American 17% Asian/Pacific Islander 0%	NR	Physical activity questionnaire 13.0 (24.0)
	control	30	55.1 (7.6)	In situ (10%) Stage I (27%) Stage II (47%) Stage IIIA (17%)	29.4 (7.4)	77.2 (20.4)	Post-menopausal	3.3 (2.6)	White 90% African American 7% Asian/Pacific Islander 3%	NR	Physical activity questionnaire 12.0 (20.0)
	Study arm	Sample Size Experimental/ control	Age mean (SD)	Disease stage (%) In situ Stage I Stage II Stage IIIA	BMI mean (SD)	Weight (kg) mean (SD)	Menopausal status *Pre-menopausal *Post-menopausal	Time since diagnosis	Ethnicity (%)	Pedometer average, steps/d	Physical activity
	Experimental	37	56.5 (9.5)	In situ -11% Stage I -54% Stage II -27% Stage IIIA -8%	30.4 (6.0)	NR	Post-menopausal	3.6 (2.2)	Non-Hispanic white 84% African American 16% Asian/Pacific Islander 0%	5145 (2,312)	-Physical Activity Questionnaire 13.0 (24.0) -Daily Activity Log, min/wk. recreational exercise 30.0 (41.1)
	control	38	55.1 (7.7)	In situ -11% Stage I -27% Stage II -46% Stage IIIA -16%	30.1 (7.4)	NR	Post-menopausal	3.3 (2.6)	Non-Hispanic white 84% African American 11% Asian/Pacific Islander 3%	5342 (2,744)	-Physical Activity Questionnaire 12.0 (20.0) -Daily Activity Log, min/wk. recreational exercise 11.3 (24.8)
	Experimental	37	56.5 (9.5)	In situ -11% Stage I -54% Stage II -27% Stage IIIA -8%	30.57 (5.95)	81.28 (16.98)	Post-menopausal	3.6 (2.2)	Non-Hispanic white 84% African American 16% Asian/Pacific Islander 0%	5.145 (2.312)	-Physical Activity Questionnaire 13.0 (24.0) Daily activity log (min/week- recreational exercise) 30.0 (41.1)
	control	38	55.1 (7.7)	In situ -11% Stage I -27% Stage II -46% Stage IIIA -16%	29.74 (7.27)	78.4 (20.01)	Post-menopausal	3.3 (2.6)	Non-Hispanic white 84% African American 11% Asian/Pacific Islander 3%	5.342 (2.744)	-Physical Activity Questionnaire 12.0 (20.0) -Daily activity log (min/wk. recreational exercise) 30.0 (41.1)
	Experimental	36	56.4 (9.5)	In situ -11% Stage I -56% Stage II -25% Stage IIIA -8%	30.4 (6.0)	81.0 (16.8)	Post-menopausal	3.6 (2.2)	Non-Hispanic white 83% African American 17% Asian/Pacific Islander 0%	5,083 (2,312)	-Physical Activity Questionnaire 13.0 (24.0) -Daily activity log (min/wk. recreational exercise) 30.0 (41.1)
	control	32	55.6 (7.7)	In situ -13% Stage I -25% Stage II -44% Stage IIIA -19%	30.1 (7.4)	79.3 (21.3)	Post-menopausal	3.3 (2.6)	Non-Hispanic white 90% African American 7% Asian/Pacific Islander 3%	5,624 (2,744)	-Physical Activity questionnaire 12.0 (20.0) - Daily activity log (min/wk. recreational exercise) 11.3 (24.8)
	Experimental	36	56.4 (9.6)	In situ -13% Stage I -25% Stage II -44% Stage IIIA -19%	30.6 (6.0)	81.3 (17.0)	Post-menopausal	3.5 (2.1%)	Non-Hispanic white 83% African American 17% Asian/Pacific Islander 0% Unknown 0%	5083 (2,313)	-Physical activity questionnaire 30.3 (41.4)
	control	31	55.4 (7.6)	In situ -11% Stage I -56% Stage II -25% Stage III -8%	29.4 (7.3)	77.3 (20.0)	Post-menopausal	3.1 (2.4%)	Non-Hispanic white 87% African American 6% Asian/Pacific Islander 3% Unknown 3%	5661 (2,740)	-Physical activity questionnaire 11.9 (31.3)
	Study arm	Sample Size Experimental/ control	Age mean (SD)	Disease stage (%) In situ Stage I Stage II Stage IIIA	BMI mean (SD)	Weight (kg) mean (SD)	Menopausal status *Pre-menopausal *Post-menopausal	Time since diagnosis	Ethnicity (%)	Pedometer average, steps/d	Physical activity
	Experimental	30	53 (11)	Stage I (33%) Stage IIa (37%) Stage IIb (20%) Stage IIIa -10%	25.9 (2.8)	74.0 (9.4)	NR	NR	NR	NR	physical performance assessment by 12min Walk Test (12MWT) 799.6 (81)
	Control	32	51 (11)	Stage I (47%) Stage IIa (25%) Stage IIb (22%) Stage IIIa -6%	26.0 (3.3)	73.0 (10.5)	NR	NR	NR	NR	-physical performance assessment by 12min Walk Test (12MWT) 814.4 (88.5)
	Experimental	37	56.5 (9.5)	In Situ 11% Stage I 54% Stage II 27% Stage IIIA 8%	30.4 (6.0)	NR	Post-menopausal	3.6 (2.2)	Non-Hispanic white 84% African American 16% Asian/Pacific Islander 0%	5.145 (2.312)	Physical Activity Questionnaire- 13.0 (24.0) -Daily Activity Log2 30.0 (41.1)
	control	38	55.1 (7.7)	In situ 11% Stage I 27% Stage II 46% Stage IIIA 16%	30.1 (7.4)	NR	Post-menopausal	3.3 (2.6)	Non-Hispanic white 84% African American 11% Asian/Pacific Islander 3%	5.342 (2,744)	-Physical Activity Questionnaire 12.0 (20.0)
	Experimental	76	55 (8)	NR	29.8 (6.2)	79.5 (17.1)	Pre & post-menopausal	54.2 (54.6)	NR	NR	-Physical activity measured by accelerometer (GT3X, Actigraph, Pensacola, FL)
	control	76	55 (8)	NR	29.8 (6.2)	79.5 (17.1)	Pre & post-menopausal	54.2 (54.6)	NR	NR	-Physical activity measured by accelerometer (GT3X, Actigraph, Pensacola, FL)
	Experimental	263	MEAN (range) 52.3 (36-68)	NR	NR	NR	Pre & post-menopausal	NR	NR	NR	*Cardiorespiratory fitness was tested by a 2-km walk test 18.6 (2.0) *Mean self-reported physical activity, MET-hr./wk. 27.40 (16.54)
	control	237	MEAN (range) 52.4 (35-68)	NR	NR	NR	Pre & post-menopausal	NR	NR	NR	*Cardiorespiratory fitness was tested by a 2-km walk test 18.4 (1.8) *Mean self-reported physical activity, MET-h/wk. 26.37 (15.69)
	Study arm	Sample Size Experimental/ control	Age mean (SD)	Disease stage (%) In situ Stage I Stage II Stage IIIA	BMI mean (SD)	Weight (kg) mean (SD)	Menopausal status *Pre-menopausal *Post-menopausal	Time since diagnosis	Ethnicity (%)	Pedometer average, steps/d	Physical activity
	Experimental	16	47.5 (7.1)	NR	NR	NR	NR	NR	white 70% Black 12% Asian 12% Native American 6%	NR	NR
	control	8	61.8 (8.1)	NR	NR	NR	NR	NR	white 88% Black 12% Asian 0% Native American 00%	NR	NR
	Continuous	9	44.1 (4.6)	NR	30 (3.4)	75.4 (10.9)	Post-menopausal	NR	NR	NR	NR
	Interval	8	44.1 (4.6)	NR	29.9 (2.8)	75.4 (6.6)	Post-menopausal	NR	NR	NR	NR
	control	6	44.1 (4.6)	NR	30.9 (4.07)	81.7 (12.3)	Post-menopausal	NR	NR	NR	NR

## Discussion

This systematic review summarized the evidence on the effects of aerobic exercise interventions (especially walking as the most frequently investigated approach) in BC survivor women. The results showed that aerobic exercise was superior to usual care in enhancing the physical activity, QoL, and sleep parameters. However, weight and inflammatory markers did not show any remarkable difference between the two groups.

Moderate intensity aerobic exercise is associated with increased short-term physical activity levels among BC survivors, especially in patients who did not follow regular exercise before (Irwin et al., 2008; Matthews et al., 2007). However, Saarto et al., (2012) reported no significant difference between the exercise and control groups. This is explained by the considerable motivation and healthy lifestyle in the control group patients, which exerted a ceiling effect on the ability to detect the exercise benefits.

The observed increase in physical activity levels significally correlates with QoL improvement. The results of Murtezani et al., (2014) confirmed the causative relationship between moderate intensity aerobic exercise and QoL improvement. These results are supported by previous studies on the efficacy of aerobic exercise in improving the overall QoL (Burnham., 2002; McNeely et al., 2006). This improvement may be due to reducing anxiety and increasing energy in addition to counteracting some negative effects of cancer treatment (Jones et al., 2013; Meneses-Echávez et al., 2015; Murtezani et al., 2014). 

Campbell et al., (2017) found that aerobic exercise ameliorated the resulting cognitive impairment from the psychological burden and cancer therapies. The results of this study come in accordance with a previous meta-analysis of 33 RCTs, which investigated the effects of exercise in general regarding the psychological outcomes, body composition and physical fuctions (Zhu et al., 2016). It concluded that exercise was beneficial for BC survivors because it improved the QoL, body configuration, and muscle strength, decreased serum levels of insulin, IGF-II, and IGFBP-1, and alleviated depression and anxiety. 

Roveda et al., (2016) examined the effects of exercise on the sleep pattern. They reported significant improvement of sleep disturbance among BC survivors. Another study conducted by Thomas et al., (2013) revealed positive effects for aerobic exercise on reversing the metabolic syndrome in sedentry BC survivors. Another study examined the effect of aerobic exercise in untrained women in a meta-analysis framework. It included different age groups and exercise intensities and emphasized the golden role of early exercise intervention in improving the cardiovascular outcomes in sedentary women (Zhang et al., 2017).

*Strengths of the study*

We followed the PRISMA statement during performance and reporting of this review and conducted all steps in accordance to the cochrane handbook for systematic reviews of interventions. Reasons for patient discontinuation were appropriately addressed. Moreover, study investigators used as intention to treat approach so there would not be attrition bias. Studies included in this systematic review were low to moderate risk of bais according to Cochrane ROB assessment tool. 

*Limitations of the study*

We selectively chose studies that investigated the benefits of aerobic exercise in BC survivors. We found scarce literature covering this pattern of exercise with diversity in the measured outcomes, intervention (type, intensity, and duration), and the used assessment methods. Therefor, the extracted data were not sufficient for subsequent pooled analysis. Future studies are recommended to study the effects of aerobic exercise in BC survivors for longer durations with including much important outcomes, such as inflammatory markers, sleep pattern, and other cancer contol measures.

*Clinical Implications*

The review findings supports that aerobic exercise intervention is beneficial to BC survivor women. It improves fatigue associated with BC by improving levels of physical activity, as well as QoL. It also enhances the stability of sleep among BC survivors (Matthews et al., 2007; Murtezani et al., 2014; Roveda et al., 2016). 

*Key Practice Points*

* Aerobic exercise should be recommended in the rehbailitation regimen of BC survivors.

* The intensity and duration of exercise should be personalized to each patient and further research is needed in this regard.

In conclusion, despite the aforementioned limitations, there is evidence that aerobic exercise (mainly in the form of walking) was associated with beneficial outcomes in BC survivor women; It improved the Qol and alleviated the symptoms of depression and anxiety. Moreover, it enhanced the physical functioning and sleep pattern. 

*Abbreviations*

BC: Breast Cancer, CRP: C - reactive protein, QoL: Quality of Life.
